# Change of home visit frequency by public health nurses predicts emergency escorts for psychiatric patients living in the community: A retrospective medical record review

**DOI:** 10.3389/fpubh.2023.1066908

**Published:** 2023-02-09

**Authors:** Meng-Chieh Wu, Chia-Chun Hung, Su-Chen Fang, Tony Szu-Hsien Lee

**Affiliations:** ^1^Department of Health Education and Health Promotion, National Taiwan Normal University, Taipei, Taiwan; ^2^Continuing Education Master's Program of Addiction Prevention and Treatment, National Taiwan Normal University, Taipei, Taiwan; ^3^Department of Nursing, Mackay Medical College, New Taipei City, Taiwan

**Keywords:** public health nurse, home visit, case management, mental disorder, emergency escort, community

## Abstract

**Background:**

Improper or insufficient treatment of mental health illness harms individuals, families, and society. When psychiatric treatment shifts from a hospital-based to a community-based health care system, risk management is essential to the provision of effective care.

**Objective:**

We examine whether an upgrade in home visit frequency of psychiatric patients as identified by public health nurses can predict the subsequent need for emergency escort services for medical treatment.

**Design:**

A 2-year retrospective medical record review.

**Setting(s):**

A district of New Taipei City in Taiwan.

**Participants:**

A total of 425 patients with a diagnosed mental health illness cared for through home visits by public health nurses from January 2018 to December 2019.

**Methods:**

We accessed the Ministry of Health and Welfare's psychiatric care management information system to identify a set of medical records, and analyzed these records using chi-square and regression analyses.

**Results:**

The analyses indicated that the groups experiencing the greatest need for emergency escort services were: male, 35–49 years old, with a senior high school level of education, without a disability identification card, with a schizophrenia diagnosis, and had been reported by the nurse as having progressed to a serious level. Nurses' increased frequency of home visits (an indicator that the patient's overall condition was worsening) and nurses' reports of increased severity of problems were significant predictors of the need for emergency escort services.

**Conclusions:**

The nurses' adjustment of visit frequency based on the results of the visit assessment predicts the need for emergency escort services for mental patients. The findings support not only the professional roles and functions of public health nurses, but also the importance of strengthening psychiatric health community support services.

## Background

Mental and addictive disorders affect more than a billion people (1). Individuals, families, and society may be subject to daily burdens and serious loss when people with mental illness do not receive proper treatment. In Taiwan, people with mental disorders have a higher mortality rate than the general population (2). The number of outpatients and inpatients (including emergency room visits) is rising every year, as are healthcare costs (3–8). The damage caused by mental illness to the social life and productivity of the patient is huge. Mental pain can be worse than physical pain; it is difficult to tolerate.

People with mental illness often experience social barriers due to stigma, culture, and negative perceptions (9). They experience higher rates of poverty and unemployment (10). Long-term isolation may cause the patient's social functions to deteriorate. They may lack of sense of reality, or have increased feelings of inferiority and dependence, which make it more difficult to recover (11). After the de-institutionalization movement in United States in the 1970's, society paid more attention to psychiatric rehabilitation and recovery (12, 13). In Taiwan, by 2007, the Mental Health Act Amendment encourage patient to return and stay in the community and the primary goal is supporting the patients to live normally in the community (14).

Outpatient care is the most common treatment for many mental health problems. However, patients with mental illness who live in the community may relapse at any time with the sudden onset of severe psychotic symptoms, impaired mental function, fluctuating symptoms, or the inability to make accurate judgments, all of which bring an increased risk of self-harm as well as of suicide (15–17). If a patient is in danger of harming him or herself or others, a consultation with a mental health professional may be needed to determine whether the patient needs the more intensive care that comes with inpatient treatment. The longer a severe mental illness goes untreated, the more damage the illness can cause (18, 19).

Public health nurses play a major role in community mental health visiting services for patients with mental disorders in Taiwan. Public health nurses need to be good communicators and critical thinkers, and they have to be able to manage multiple priorities, especially in preventive and therapeutic psychiatry (20–24). A quality therapeutic relationship between the healthcare provider and a patient with mental illness is critical to successful treatment. It is also a reliable predictor of clinical outcomes (25). Patients' understanding of their own status through public health nurses can increase their awareness of their condition, which can effectively allow them to recognize and accept the progress of their disease, and help them to seek medical care when needed to reduce damage and stabilize their condition (26, 27). Furthermore, nurse-delivered interventions, such as case management, telephone and in-person supportive contact, problem-solving, and basic psychoeducational counseling can improve patients' depression, anxiety, physical and social functions, medication compliance, and life satisfaction (28, 29).

The literature shows that compulsory treatment reduces the number of days of hospitalization and frequency of psychiatric readmission, and it increases usage of community services and treatment adherence (30). Involuntary treatment and care that involves the use of coercive measures is a common psychiatry practice, both for protecting the safety of patients and the community, and for therapeutic reasons (31, 32). Such measures are essential in preventing a person at risk from endangering themselves or causing harm to others. Although coercive measures have always been a psychiatric treatment tool, the ethical dilemma between patient dignity and the use of therapeutic coercive measures remains a contentious issue in mental health research and practice (33, 34).

The Ministry of Health and Social Welfare in Taiwan established legal guidelines to regulate the compulsory treatment of people with mental disorders (35). Compulsory treatment consists of a standard operating procedure for escorting psychiatric patients living in the community to medical treatment when it is needed. Through the trilogy of *identify, coordinate*, and *escort to hospital*, community members can assist people with suspected mental illness or psychiatric patients who have harmed themselves or others in seeking medical support. After obtaining supporting evidence from a public health nurse that the notified person is a mental patient, the next steps are to clarify the level of risk to the patient or community, identify how best to assist, and determine whether an emergency escort to a medical facility is required.

### Case management

The literature shows that nurse-led case management of mental health promotion interventions are feasible, acceptable, and have sustained impact. Nurse-led care can effectively improve patient outcomes, reduce the use of expensive health care, and enhance clinical practice among home care providers (36). Case management originated in the United States in the 1950's and was first used with cases involving mental health problems. It was then mostly used to coordinate community services in caring for patients with high cost high-risk health problems (37). The case management model is an effective method in a primary care setting to improve medication adherence in patients with mental disorders (38, 39). It can reduce emergency room visits, readmissions, and the length of hospital stays (40, 41), and it improves clinical indicators and the satisfaction of the patients (42). Frontline medical staff such as public health nurses are often asked to manage cases. They can have a vital role in education, identification of health problems, and modification of health behavior through home visits.

In Taiwan's public health system, case management is one of the main tasks of the nursing staff. The purpose of case management is to improve the quality of care and clinical outcomes of the case, and to reduce inappropriate admissions, as well as the time spent in crisis and the length of the hospital stay. Good case management can also reduce resource utilization while ensuring the continuity of care and cost control. Thus, quality of care, length of hospital stay, resource utilization, the necessity of continuous care, and cost are all important indicators for the evaluation of case management (15, 43–45).

Little attention has been paid to whether home visits can predict the patient's need for an emergency medical escort (any visit to an emergency room accompanied by police or emergency medical technician). Several studies have identified specific risk factors for psychosis such as positive symptoms (46, 47). Rapid assessment of a person's mental state can be carried out in four dimensions: affect, behavior, cognition, and drive (48, 49). When public health nurses visit a patient in the community, they focus on risk assessment, not just symptom assessment. They are often exposed to complex and controversial emergency mental health issues. They must rely on their own competence to identify the early warning signs of mental illness in conjunction with current symptoms, and then they need to be able to take action to reduce a patient's likelihood of self-harm, or manage aggressive or violent behavior. In this way, public health nurses can reduce the need for an emergency medical escort and emergency room visits. In this study, we examine whether the change in a patient's illness severity as identified through home visits by public health nurses can predict the need for subsequent emergency medical escort services.

## Method

In Taiwan, Community Care Services for Psychiatric Patients is a specific measure taken by the government to build a social safety net. The Ministry of Health and Welfare promulgated “Key Points of Community Home Visits for People with Mental Health Disorders,” which specifies that people with mental health illness must be enrolled in the Psychiatric Care Information Management System for care management. Public health nurses act as case managers and provide home visits combined with telephone interviews. Public health nurses can adjust visit frequency according to the grade of care from monthly to annually.

The Research Ethics Committee at the National Taiwan Normal University (202106HS013) and the Institutional Review Board of New Taipei City (1116360736) reviewed and approved the study protocol.

We used secondary data from the Psychiatric Care Information Management System to evaluate patient care by public health nurses. This study is not registered for clinical trials. Patients include those discharged from hospitals, released from prison, and those assigned by the health department supervisor. After visiting a patient who is newly assigned through the Psychiatric Care Information Management System, public health nurses key in nursing records including the patient's personal information, medical history, and health status. We used these records to composite our study sample.

### Data refinement and analysis

We identified a total of 500 patients who received home visiting services by public health nurses due to a mental health illness in a district of the New Taipei City in Taiwan from 2018 to 2019 in the Psychiatric Care Information Management System. Each patient was retrospectively reviewed for medical records in 2 years. We excluded 33 patients from the study because they either moved out of the city or contracted another disease in 2018–2019. We excluded an additional 21 patients with incomplete records. A total of 425 patients were included in the study.

We focused on nine independent variables: gender, age, education, diagnosis, degree of disability, classification of major illness, the nurse's reported change in the intensity of care for the patient (visit frequency), and the nurse's reported change in the severity of illness of the patient. We defined the outcome variable, emergency escort, as any visit to an emergency room accompanied by police or emergency medical technician, including an observation stay that did not result in admission. We defined the severity of mental health illness according to Taiwan's Mental Health Act: patients who demonstrate strange thoughts and unusual behavior that are out of touch with reality so that they cannot handle their own affairs, and whose diagnosis is confirmed by a psychiatrist. which is categorized from. The grade is determined based on five dimensions: positive symptoms, life dysfunctions, psychological disturbance, patient care by primary caregivers, and medical adherence. Each dimension can be scored from 1 (none) to 4 (very serious). There are four home visit frequency based on the total score: 0 to 4 (grades 1), 5 to 8 (grade 2), 9 to 14 (grade 3), and 15 to 20 (grade 4). When scores assessed increase or decrease, the public health nurse change the home visit frequency to higher or lower grades, respectively.

We used SPSS 22.0 statistical software to manage the data and perform the statistical analyses. Missing data were all excluded from the analysis. We applied Chi-square tests to examine the bivariate associations related to the use of emergency medical escort services. *F*-test for logistic regression is significant which fits the assumption. Thus, we used multiple logistic regressions to predict the need for an emergency medical escort through social demographics and the records provided by the public health nurses.

## Results

### Sociodemographic characteristics

Of the 425 patients, 238 (56%) were female and 187 (44%) were male. Their ages ranged from 16 to 86 years, with a mean of 47.6 years old. 44% had only a junior school education or below. Schizophrenia was the most prevalent diagnosis (48%), followed by bipolar affective psychosis (33%). A total of 28, 32, and 7% were assessed as mild, moderate and severe functional impairment, respectively according to the International Classification of Functioning, Disability, and Health (ICF). The most severe cases were mainly diagnosed with schizophrenia.

### Use of emergency medical escorts by demographics

The distribution of use of emergency medical escort services was as follows: a total of 57 emergency medical escorts for 33 (7.8%) persons, of which 19 (4.5%) were escorted to an emergency room once, eight (1.9%) went twice, four (0.9%) went thrice, one (0.2%) went four times, and one (0.2%) went six times. In terms of gender, 19 males (57.6%) and 14 females (42.4%) required emergency medical escorts. One person (3%) was under 19 years old, eight people (24.2%) were 20–34 years old, 18 (54.6%) people were 35–49 years old, and six (18.2%) were 50–64 years old. No one was over 65 years old. For education, 11 (30%) had a junior high school education, 15 (45.6%) had completed high school, and seven (21.2%) completed college. Thirteen persons (39.4%) were classified as disabled, and 18 (54.5%) were classified with major illness. Of the patients who required emergency medical escorts, 17 (51.5%) were diagnosed with schizophrenia with 26 visits to the emergency room, and 10 (30.3%) had bipolar disorder with 19 visits. Their diagnoses were as classified by The International Statistical Classification of Diseases and Related Health Problems 9th Revision (ICD-9). No significant difference in emergency escort use was found for gender, age, education, diagnosis, degree of disability, or classification of illness.

### Prediction of the need for an emergency medical escort

We found significant differences between groups that differed in terms of the nurse's reported change in intensity of case management care (worsening assessment) (32.4%) (χ^2^ = 36.53, *p* < 0.05), and for the nurse's reported severity of illness (31.8%) (χ^2^ = 18.74, *p* < 0.05) (see Table 1).

**Table 1:** The relationship between sociodemographic variables of people with mental illness in a district of a city and use emergency medical escort from 2018 to 2019.

**Variable**	**No emergency medical escort**		**Emergency medical escort**		**Total**	
	***n***	**%**	***n***	**%**	***n*** **(%)**	χ^2^
**Gender**						2.68
Male	168	43%	19	58%	187 (44%)	
Female	224	57%	14	42%	238 (56%)	
**Age**						1.70
Under 34	64	16%	8	24%	72 (17%)	
35–49	165	42%	11	33%	176 (41%)	
Over 50	163	42%	14	43%	177 (42%)	
**Education**						1.73
Junior high or below	177	45%	11	33%	188 (44%)	
High school	146	37%	15	46%	161 (38%)	
College and above	69	18%	7	21%	76 (18%)	
**Diagnosis**						0.20
Schizophrenia	187	48%	17	52%	204 (48%)	
Bipolar disorder	132	34%	10	30%	142 (33%)	
Others	73	18%	6	18%	79 (19%)	
**Disability identification**						1.48
No	126	32%	13	39%	139 (33%)	
Mild	111	28%	9	27%	120 (28%)	
Moderate	129	33%	8	25%	137 (32%)	
Severe and profound	26	7%	3	9%	29 (7%)	
**Classification of major illness**						0.00
No	180	46%	15	45%	195 (46%)	
Yes	212	54%	18	55%	230 (54%)	
**Change of care grade**						36.53^**^
Reduced	25	6%	12	36%	37 (9%)	
Retained	242	62%	10	31%	252 (59%)	
Increased	125	32%	11	33%	136 (32%)	
**Serious mental patient**						18.74^**^
Yes	15	4%	7	21%	22 (5%)	
No	377	96%	26	79%	403 (95%)	

** *p* < 0.01.

Regardless of the type of visit by the public health nurse (home or telephone calls to patients or their family members), these groups did not have a statistically significant difference in the use of emergency medical escort services. According to the multiple logistic regression analysis, an increase in the intensity of case management care (OR = 6.99; 95% CI: 1.36–35.87) or in the reported severity of illness (OR = 12.91; 95% CI: 2.06–81.06) were significant predictors of the need for emergency escort services (see Table 2).

**Table 2:** Prediction of emergency medical escorts for people with mental illness in a district of a city located in North Taiwan from 2018 to 2019 using the logistic regression.

**Varialbe**		**Emergency escort**		
	**n (%)**	**(n)**	**(%)**	**OR**	**95%CI**
**Change of care grade**					
Grade increased	136 (32%)	11	8.1%		
Grade reduced	37 (9%)	12	32.4%	6.99^*^	1.364~35.872
Grade retained	252 (59%)	10	4.0%	0.44	0.076~2.523
**Serious mental patient**					
No	403 (95%)	26	6.5%		
Yes	22 (5%)	7	31.8%	12.91^**^	2.056~81.062

^*^p < 0.05; ^**^p < 0.01.

## Discussion

This is one of the few studies to examine the relationship between emergency medical escort and home visiting services by public health nurses in patient with mental disorder. The finding is consistent with our hypothesis that patients who required to increase home visiting services were associated with an emergency medical escort. This result indicated that the public health nurses play an important role for the assessment patient conditions and provided crisis intervention for patients with mental disorder who live in community. This information may be of use in the development of a suitable strategy for managing these emergency services. This is consistent with a previous study showed that patients with psychosis attempts that increased risk for involuntary treatment in Taiwan (50). When patients who increased home visiting services indicated that the symptoms of psychosis conditions change for the worse. This suggests that public health nurse is a qualified evaluator in identifying the disease progress in early stages to prevent emergency escort to involuntary treatment or relapse of mental illness. A public health nurse with knowledge and skills and a professional manner can use telephone visits, home visits, or office visits to manage patients and to assess patient and family risk profiles. The benefit of being able to predict the need for medical escort services outweighs the cost of frequent home visits.

Community-based case management provided by nurses can improve patients' depression, anxiety, physical and social function, and improve their clinical indicators (29, 42). Mental health education in primary care settings, such as home visits and proactive strategies, can strengthen community and national mental care policies (51). Other researchers have pointed out that expanding home visits to high-risk groups improves medical comorbidities, and reduces readmissions within the year, alcohol use disorders, and bipolar disorder (52) and they can significantly lower medical expenditures and reduce the utilization rate of emergency departments and hospitalization (53). In Japan, the spread of home-visit care services for people with mental illness is now growing rapidly, and research shows that community psychiatric care can improve quality of life for patients and their families (54). High-intensity case management intervention requires a small caseload, regular follow-up, and multidisciplinary/interorganizational care planning. Community-based psychosocial interventions may have a favorable influence on clinical outcomes, including improved functioning; reduced hospital admissions, length of stay, emergency department visits, and health care costs; and increased patient motivation, self-management, care coordination, and efficacy of referral (55, 56). Public health nurses play a vital role in community mental health care. Patients with complex care needs can benefit the most (22, 57). When a public health nurse finds that the risk of a sudden onset of psychotic behavior is increasing, adjusting the visit frequency and early intervention are essential.

The finding indicated that to identify symptoms early and change the level of care can be integrated with different but complementary detection approaches across the community, primary, and secondary care (58). Coordinated care can improve patient outcomes during transitions, longitudinal high-risk care management, and unplanned acute episodic care (59). Health care administration and social welfare departments need to cooperate with family members to provide active public mental health care services, prevent mental illness, reduce the occurrence of community nuisance cases, reduce the burden on caregivers, and improve care satisfaction (60–62).

A study by Danish academics identified predictors of high psychiatric emergency room usage, which included mental illness, substance abuse, and shelter residence (63). However, the notification process of patients with severe mental illness is cumbersome. It is difficult to identify just who qualifies as a person with severe mental illness because psychiatrists may differ in opinion. Omissions or ignored reports by psychiatrists are possible, and more research is needed.

Contrary to previous research (64, 65), we found no association with gender or age and use of emergency escort services. However, male and younger patients had the highest proportion of emergency escorts, which is in line with studies conducted in Ethiopia and Australia (66, 67). Public health nurses need greater awareness and vigilance to identify and proactively discover high-risk groups to take precautions.

### Limitations

There are a few limitations. This study adopts a retrospective medical record design and used the Psychiatric Care Information Management System database for analysis. Public health nurses manually entered the content of the database. There may be misplaced, inconsistent, or insufficient data entry and the authenticity of the data cannot be verified. Data used in this study were confined to a particular district in one city, which may limit its representativeness to the rest of the population and limit utility of inference based on the results.

## Conclusions

For people with mental illness living in the community, changes in the intensity of care or in the severity of illness as reported by a public health nurse may have predictive power for the use of emergency medical escort services. This result supports the value of home visits and psychiatric care in the community. Public health nurses play an important role in community mental health care. When a public health nurse finds that the risk of the sudden onset of psychotic behavior is increased, it is necessary to take early intervention, such as assurance of medical adherence or schedule an inpatient visit as soon as possible.

## Data availability statement

The raw data supporting the conclusions of this article will be made available by the authors upon reasonable request.

## Author contributions

M-CW and TS-HL designed the study and drafted the manuscript. M-CW collected and cleaned data. M-CW and C-CH performed the statistical computations and prepared the results. C-CH and TS-HL provided intellectual interpretation and conclusion. All authors drafted parts of the manuscript and approved the final manuscript.
